# An unnatural enzyme with endonuclease activity towards small non-coding RNAs

**DOI:** 10.1038/s41467-023-39105-0

**Published:** 2023-06-24

**Authors:** Noreen Ahmed, Nadine Ahmed, Didier A. Bilodeau, John Paul Pezacki

**Affiliations:** Department of Chemistry and Biomolecular Sciences, University of Ottawa, Ottawa, Ontario K1N 6N5 Canada

**Keywords:** Biocatalysis, Synthetic biology, Protein design, Enzymes, RNA

## Abstract

Endonucleases are enzymes that cleave internal phosphodiester bonds within double-stranded DNA or RNA and are essential for biological functions. Herein, we use genetic code expansion to create an unnatural endonuclease that cleaves non-coding RNAs including short interfering RNA (siRNA) and microRNAs (miRNAs), a function that does not exist in nature. We introduce a metal-chelating unnatural amino acid, (2,2′-bipyridin-5-yl)alanine (BpyAla) to impart endonuclease activity to the viral suppressor of RNA silencing protein p19. Upon binding of copper, the mutant p19-T111BpyAla displays catalytic site-specific cleavage of siRNA and human miRNAs. Catalysis is confirmed using fluorescence polarization and fluorescence turn-on. Global miRNA profiling reveals that the engineered enzyme cleaves miRNAs in a human cell line. The therapeutic potential is demonstrated by targeting miR-122, a critical host factor for the hepatitis C virus (HCV). Unnatural endonuclease function is shown to deplete miR-122 levels with similar effects to an antagomir that reduces HCV levels therapeutically.

## Abstract (web summary)

Endonucleases play crucial roles in various biological processes but endonucleases that target small non-coding RNAs have not been reported. Here, the authors combined the metal binding non-canonical amino acid BpyAla and a high affinity binder to engineer a catalyst that degrades small non-coding RNAs.

## Introduction

Endonucleases catalyze the cleavage of internal phosphodiester bonds in RNA and DNA leading to scissile rupture of genetic material or their transcription products. Endonuclease function in many organisms is involved in DNA damage repair mechanisms, restricting gene expression and antiviral responses to infection in bacteria^1^. Endonucleases that target RNA substrates are involved in mRNA splicing, RNA silencing pathways, maturation of t-RNAs, and other non-coding RNAs^2–5^. Adapting and engineering these natural enzyme catalysts has led to cornerstone biotechnologies from recombinant DNAs to gene editing technologies^6–8^.

Short non-coding RNA molecules (ncRNAs) such as microRNAs (miRNAs) have important regulatory roles^9,10^. miRNAs have been identified to play critical roles in gene expression regulation during transcriptional processes^11^, as well as different biological and biochemical processes ranging from cellular metabolism to immunity^10,12,13^. As products of the RNA silencing pathway, miRNAs are usually 21–25 nucleotides in length^9,10,14^. They exert their function by targeting specific messenger RNAs (mRNA) for degradation or translational repression^10,13,15^. Also, miRNA-based regulation has been linked to disease progression, which makes them interesting targets for protein-based therapeutics^10,16^. While there do exist natural suppressors of RNA silencing^17–20^, these typically bind and sequester RNA intermediates. No naturally occurring endonuclease exists that catalyzes the specific degradation of either siRNAs or miRNAs.

Designing endonuclease enzymes, and indeed any enzyme, based on substrate-binding scaffolds (e.g., proteins, aptamers, catalytic antibodies) have had limited success because the product closely resemble the bound transition state, causing product inhibition which prevents enzymatic turnover^21–25^. Thus, the product needs to be significantly different from the substrate and the transition state structures for the designed endonuclease enzyme to be successful. The binding protein needs to retain enough selectivity to its substrate, that once the preferential binding characteristics are lost, the product is released, and product inhibition is avoided^25^.

**Fig. 1:**
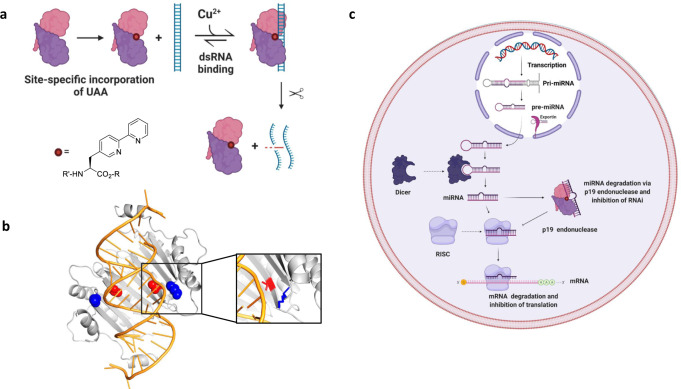
Incorporation of BpyAla in 2 sites in the p19 dimer. **a** Incorporation of BpyAla site-specifically into the VSRS protein p19 dimer introduces a metal binding site. Here we used copper to bind to BpyAla. If the metal can cleave the phosphodiester linkage within the p19 substrate, then it should induce site-specific strand cleavage of the bound siRNA or miRNA. Cleavage can occur on either strand. Created with BioRender.com. **b** Crystal structure of the p19 dimer with K67 (blue) and T111 (red) highlighted PDB: 1RPU. **c** Overall Schematic representation of the potential disruption of the RNA silencing pathway upon treatment with the engineered p19 endonuclease. Created with BioRender.com.

To engineer a new endonuclease capable of specifically targeting miRNAs, we started with the Tombusvirus p19 viral suppressor of RNA silencing (VSRS), which naturally binds and sequesters small RNA duplexes with picomolar affinity, thereby preventing the activation of the RNA silencing pathway^17,18,26–30^ (Fig. 1a, c). p19 binds RNA sequences with size selectivity^26,27^. Rational design and protein engineering of the p19 VSRS has allowed the development of biotechnological tools with diverse applications^17,27,28,31–37^. p19 has proved to be a potent inhibitor of small RNA function, which opens the door to further engineering the protein to exhibit “super suppressor” activity with added endonuclease functionality^17,33–36^. Previously, it has been shown that using the pioneering technologies of genetic code expansion in combination with unnatural amino acids (UAAs), different organisms can code for more than the 20 canonical amino acids allowing for the incorporation of unique chemical side chains into the desired location in a protein of interest^38–40^. It has been shown that the attachment of metal chelating molecules such as Ethylenediaminetetraacetic acid (EDTA) and phenanthroline derivatives to nucleic acid binding agents has led to oxidative and hydrolytic cleavage^41^.

In this work, we explore the potential of introducing metals site-specifically into p19. The introduction of BpyAla chelated divalent ions is utilized, leading to site-specific RNA cleavage. Here we use p19, an RNA-binding protein with high selectivity to double-stranded small RNAs of 19–25 nucleotides in length but a very low affinity for single-stranded (ssRNA) and shorter double-stranded RNA (dsRNA). We successfully engineer it to cleave its native substrates. The designed endonuclease displays multiple turnover events since the protein no longer binds the cleaved products due to the loss of preferential binding characteristics. This strategy can provide a therapeutic tool targeting miRNAs involved in disease progression, as well as showing the potential of introducing catalytic activity into proteins of interest with affinities towards nucleic acids.

## Results

### Site-specific introduction of (2,2′-bipyridin-5-yl)alanine into the p19 dimer

We introduced (2,2′-bipyridin-5-yl)alanine (BpyAla)^42,43^, within the RNA-binding pocket of p19 to insert a catalytic site (Fig. 1b). The UAA bearing a bipyridyl moiety, provides an unnatural side chain capable of binding transition metal ions such as copper and iron that can catalyze the cleavage of the phosphodiester backbone of nucleic acids^42,44^.

We previously established the successful incorporation of Azidophenylalanine (AzF) into p19 at positions K67 and T111, in which full-length expression of the mutants and successful irreversible photo-cross-linking to their canonical substrates was achieved to produce cross-linked products with intermediates of the RNA silencing pathway^37^, with potential applications to CLIP assays. These two sites are found in the binding pocket of the p19 dimer, near the phosphate backbone of the RNA (Fig. 1b). To incorporate BpyAla, amber codons (TAG) were introduced in place of K67 and T111, to determine which position would allow for optimal cleavage of the bound RNA product. We expressed p19 mutants in the presence of the evolved tRNA/aaRS pair, and the presence of 1 mM BpyAla. (Supplementary Fig. 1). Bacterial cells (BL2l (DE3)) expressing the tRNA/BpyRS pair and the mutant p19 genes were grown in Lysogency Broth (LB). The full-length expression was detected using western blot analysis under the induction of Isopropyl β-d-1-thiogalactopyranoside (IPTG) (Supplementary Fig. 1)^42^.

We have confirmed the formation of p19-T111BpyAla enzyme-copper (II) complex using inductively coupled plasma mass spectrometry (ICP-MS). ICP-MS data shows that the associated copper (II) concentrations with p19-T111BpyAla are significantly higher than that associated with the control p19-WT samples, thus confirming copper ion chelation to T111BpyAla (Supplementary Fig. 2). In addition, the UV-Vis absorption spectra of p19-T111BpyAla showed two new absorption peaks at 319 nm and 304 nm upon the addition of CuSO_4_, and a decrease in absorption at 283 nm (Supplementary Fig. 3). Spectral changes are consistent with the red-shift of π–π* transition of the incorporated bipyridyl moiety upon chelation of Cu^2+^ ion, further confirming the formation of the artificial metal-bound enzyme. These data taken together confirm the specific binding of copper to the BpyAla residue within p19-T111BpyAla.

### Small RNA cleavage using p19-T111BpyAla

**Fig. 2:**
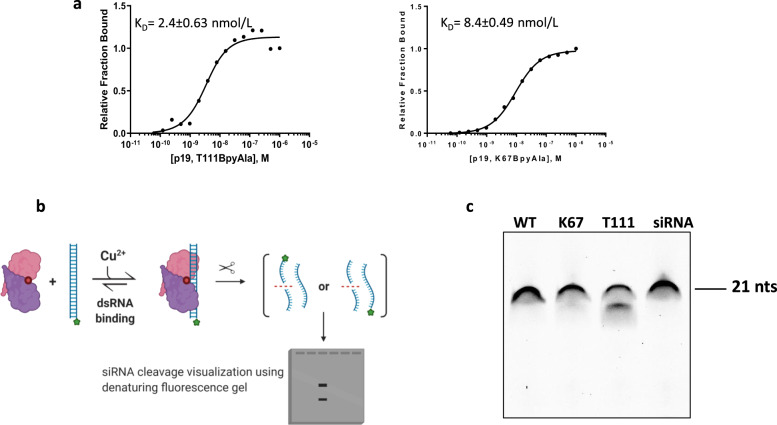
siRNA cleavage by p19-T111BpyAla mutant. **a** Binding plots for p19-T111BpyAla and for p19-K67BpyAla using electrophoretic mobility shift assay. Binding plots were constructed by varying the concentrations of the protein (0–1 µM), while maintaining the concentration of Cy3-labeled GL2 at 2 nM. **b** An overall schematic representation of the experimental strategy, in which Cy3-GL2 siRNA cleavage was assessed using a denaturing urea-PAGE gel. Created with BioRender.com. **c** 20% urea-PAGE gel was used to visualize the degradation product of Cy3-Gl2 siRNA by the engineered p19-T111BpyAla mutant in the presence of reducing agent and CuSO_4_. Gel was independently reproduced 2 times, where different purified protein stocks were used.

Next, we sought to assess the binding affinity of the mutants to a 21nts siRNA targeting Firefly luciferase (GL2) and containing Cy3 at the 5′ end of the guide strand (Fig. 2a, Supplementary Fig. 4). We determined the binding affinities of the purified recombinant mutants using an electrophoretic mobility shift assay (EMSA). The binding affinity of p19-T111BpyAla was determined to be 2.38 ± 0.63 nmol/L, while p19-K67BpyAla is 8.41 ± 0.49 nmol/L (Fig. 2a, Supplementary Fig. 4). Overall, both mutants were shown to bind siRNA with high affinity, in which the incorporated UAA has minimal perturbation to the binding functions.

To further assess the success of introducing the catalytic functionality into the selected sites in the p19 dimer, both mutants were tested for their ability to cleave Cy3 labeled 21 nucleotides long RNA duplex substrate (GL2 siRNA). The site-specific cleavage of RNA was investigated using a gel-retardation assay (Fig. 2b, c). The Cy3-tagged siRNA duplexes were incubated with p19-T111BpyAla, p19-K67BpyAla, or p19-WT, in the presence of Cu (II). The cleavage reactions were analyzed using denaturing urea-PAGE (Fig. 2b, c). The gel has revealed that only p19-T111BpyAla was able to catalyze the cleavage of the siRNA-tagged ligand leading to a lower-size band appearing on the gel. On the other hand, P19-K67BpyAla did not catalyze the cleavage of the substrate. No cleavage occurred with p19-WT under the same conditions (Fig. 2c).

### Cleavage of siRNA by p19-T111BpyAla detected using fluorescence polarization

**Fig. 3:**
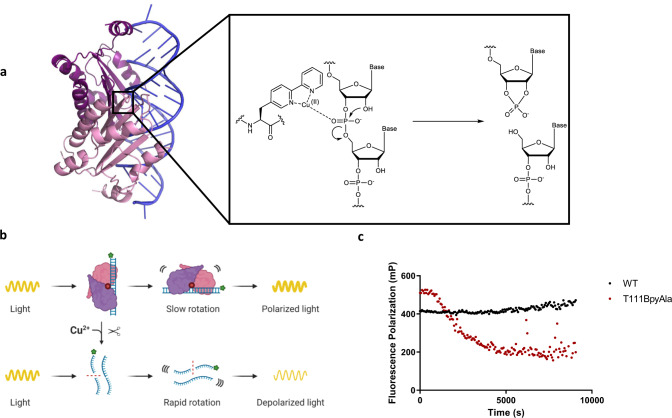
Detection of siRNA cleavage by p19-T111BpyAla using fluorescence polarization assay. **a** The overall hydrolytic RNA cleavage mechanism due to the incorporation of BpyAla into p19. **b** An overall schematic representation of the experimental strategy, in which Cy3-Gl2 siRNA cleavage was assessed using fluorescence polarization. Created with BioRender.com. **c** Fluorescence polarization assay was used to visualize the cleaving potential of p19-T111BpyAla. The assay was conducted using 5 µM p19-T111BpyAla or p19-WT and 200 nM RNA, *n* = 3, in which *n* values represent 3 independent replicates. Red dots represent p19-T111BpyAla, and black dots represent p19-WT.

Next, we sought to validate the catalytic function using fluorescence polarization assays, in which p19-T111BpyAla was incubated with a fluorescently tagged siRNA. A decrease in polarization has been observed over time, which is expected as upon cleavage and release of the substrate. The fluorescently tagged RNA is released from the enzyme, leading to faster rotation and consequently, a lower polarization signal (Fig. 3). The fluorescence polarization assay measured the degree of polarization of the fluorophore-tagged RNA and the change in fluorescence polarization as measured by the ratio of parallel and perpendicular light^45,46^. We used a Cy3-tagged double-stranded siRNA, which when unbound exhibits a low signal, however, upon being bound to the target protein, the fluorophore exhibits a higher polarization signal due to the increase in molecular mass and resultant slower rotation of the fluorophore bound to the complex^45^. When p19-T111BpyAla is combined with Cy3-siRNA, a time-dependent decrease in fluorescence polarization signal is observed (Fig. 3b, c). The decrease in fluorescence polarization signal is attributed to the cleavage of the bound siRNA molecules, leading to the release of the Cy3-siRNA from the protein complex. The release of the fragmented RNA exhibits a faster rotation^45,46^. These results confirm the hydrolytic cleavage of small RNAs due to the site-specific introduction of BpyAla into p19.

### Determination of the kinetic parameters of p19-T111BpyAla using a fluorescence turn-on assay

**Fig. 4:**
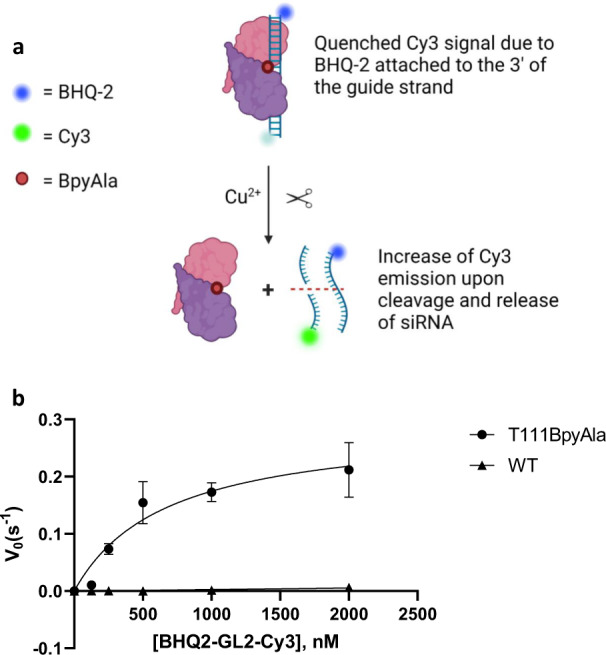
Determination of Michaelis–Menten kinetic parameters using a fluorescence turn-on assay. **a** A schematic representation of the enzymatic cleavage assay using a fluorescence turn-on approach, where if cleavage occurs an increase of Cy3 fluorescence is observed. Created with BioRender.com. **b** Steady-state cleavage velocities (MTO rates) are plotted against the concentration of substrate. 100 nM p19-T111BpyAla or p19-WT was incubated with varying concentrations of Cy3-GL2-BHQ-2 substrate to calculate the kinetic parameters at 37 °C. siRNA cleavage was monitored using fluorescence, in which initial velocities were derived from the initial slopes. Error bars represent mean values ± SD for 3 independent replicates, representing 3 independent purified protein batches. Black circles represent p19-T111BpyAla and Black triangles represent p19-WT.

Next, we sought to utilize a fluorescence turn-on assay to determine the kinetic parameters of the engineered enzyme, p19-T111BpyAla. We designed a siRNA probe containing a 3′BHQ-2 quencher on the passenger strand, while the guide strand of the siRNA contains a 3′ Cy3 fluorophore. The distance between the quencher and fluorophore is 59.7 Å, which allows efficient quenching as it is within fluorescence resonance energy transfer (FRET) distance (1–100 Å). Upon incubation of p19-T111BpyAla and Cu^2+^ ions with various, excess concentrations of the siRNA probe, an increase of fluorescence over time is observed (Fig. 4a). The increase of fluorescence is only observed with p19-T111BpyAla and not with the p19-WT protein, indicating that cleavage and release of the siRNA take place, as an increase in the Cy3 fluorescence is observed over time (Supplemental Fig. 5). The *k*_cat_ was determined to be 3.0 × 10^−3 ^s^−1^ while K_M_ was calculated to be 670 nM. The catalytic efficiency of the engineered enzyme was thus determined to be 4.47 × 10^3 ^M^−1^ s^−1^ (Fig. 4b).

### p19-T111BpyAla can cleave miRNAs isolated from hepatocellular carcinoma cell line

**Fig. 5:**
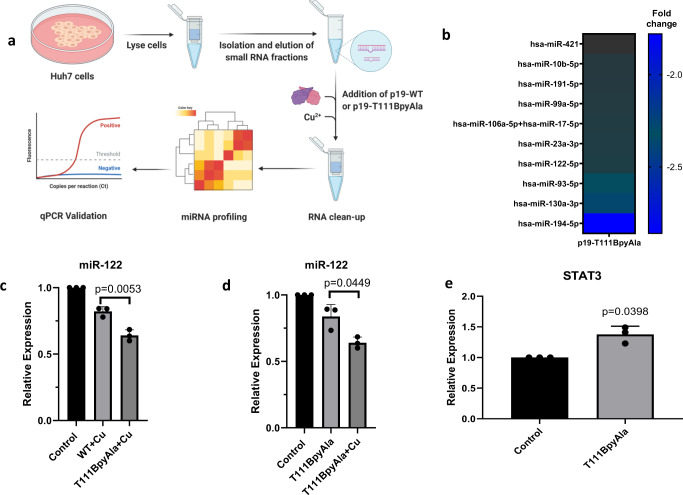
Cleavage of Huh7 miRNA samples using p19-T111BpyAla. **a** Schematic representation of the workflow for small RNA isolation for Nanostring profiling and RT-qPCR analysis. RNA used was isolated from Huh7 hepatocellular carcinoma cell line and treated in the presence of CuSO_4_. Created with BioRender.com. **b** A heat map depicting some of the significant downregulated fold changes in miRNAs upon treatment with p19-T111BpyAla relative to p19-WT treated miRNAs, *n* = 2, *n* represents 2 biological trials, where RNA is isolated from 2 independent cell passages. miRNAs with counts below 20 were eliminated from the heat map. Changes presented are more than 1.5-fold change. **c** qRT-PCR analysis of the relative miR-122 expression in p19-T111BpyAla treated samples in comparison to that treated with p19-WT in the presence of copper ions. Error bars represent the mean ± SD, *n* = 3, *n* values represent the treatment of RNA isolated from 3 different passages. **d** qRT-PCR analysis of the relative miR-122 expression in p19-T111BpyAla treated samples in the presence and absence of copper ions. Error bars represent the mean ± SD, *n* = 3, *n* values represent the treatment of RNA isolated from 3 different passages. **e** Derepression of miR-122’s downstream direct target *STAT3* upon treatment with p19-T111BpyAla, Error bars represent the mean ± SD, *n* = 3. *n* values represent the transfection of cells from 3 different passages. Unpaired two-tailed t-test was used to evaluate statistical significance.

To further demonstrate the utility of p19-T111BpyAla as an artificial endonuclease for degrading small double-stranded RNA intermediates, we examined its ability to enzymatically cleave the small RNA fractions from Huh7 human hepatoma cells. The small RNA fractions from Huh7 cells were treated with either p19-WT treatment, or p19-T111BpyAla in the presence of CuSO_4_ for 4 h at 37 °C. The treated RNA was then further purified and concentrated to ensure the removal of the proteins from the samples (Fig. 5a). To uncover the identities of the substrates for the engineered endonuclease, we conducted a miRNA profiling experiment using Nanostring technology (Fig. 5b). We isolated the small RNA fractions from Huh7 cell line and treated with either p19-WT or p19-T111BpyAla. When comparing the profile of p19-T111BpyAla to that of the wild-type protein, we identified a list of statistically significant miRNAs that are differentially downregulated compared to the p19-WT (*P* ≤ 0.05) (Fig. 5b). These data suggest that the engineered protein degrades select miRNAs and gives rise to an amplified effect on the small RNA milieu as compared to that of its WT counterpart that can only sequester miRNAs but not degrade them (Supplementary Table 1). Interestingly, miR-122-5p was observed to be downregulated by 2.0 folds when treated with p19-T111BpyAla in comparison to treatment with WT. miR-122 is one of the miRNAs of highest abundance in the liver. Other miRNAs are identified in this screen, however, p19’s affinity to these identified has not been previously examined. It is possible that p19-T111BpyAla can potentially exhibit preferential binding to these miRNAs based on the positioning of the mismatched bulges in their secondary structure, which could give rise to enhanced binding, and thus preferential cleavage as previously examined with miR-122^27^. However, further structure-function analysis needs to be performed to validate the preferential cleavage pattern obtained from the Nanostring screen. p19 is known to exhibit a high degree of size selectivity, and some degree of sequence dependence when it comes to the binding of miRNAs^27^. miR-122 represents more than 70% of the miRNA pool in hepatoma cells^47,48^, and due to the instrumental role it exhibits in hepatitis C virus replication (HCV)^48^, we decided to proceed with examining the effects of T111BpyAla on miR-122 specifically.

Next, we validated the Nanotring results by measuring the levels of miR-122, upon treatment with the mutant in the isolated samples using RT-qPCR. From Fig. 5c, it is evident that there is an observed decrease in the levels of miR-122 in p19-T111BpyAla samples, relative to treatment with p19-WT in the presence of copper, which aligns with the results from the Nanostring profiling (Fig. 5b). We attribute this decrease to the degradation of miR-122 by the mutant enzyme, as an enhanced degradation/decrease in miR-122 levels is only observed upon the introduction of copper ions (Fig. 5d). Derepression of a miR-122 direct target *STAT3* was observed upon treatment with p19-T111BpyAla (Fig. 5e). These results together aid in demonstrating biocatalysis in a complex sample. In addition, there is no enhanced binding observed towards miR-122 by p19-T111BpyAla in comparison to the p19-WT when analyzed by EMSA (Supplemental Fig. 6).

For this study, we chose to next focus on miR-122 as it is a highly expressed miRNA in hepatic cells and is known to play a major role as a proviral host factor in the propagation of HCV, a positive-sense single-stranded RNA virus of the family Flaviviridae^38–40^. HCV is known to be a causative agent of hepatitis and hepatocellular carcinoma and lymphomas in humans. It is well established that HCV uniquely requires the liver-specific miR-122 for its replication. It exerts a positive effect on HCV RNA levels by binding directly to a site in the 5′-UTR of the viral RNA, and contributes to the stability, translation, and replication of the viral RNA^49–51^.

### p19-T111BpyAla represses HCV replication

**Fig. 6:**
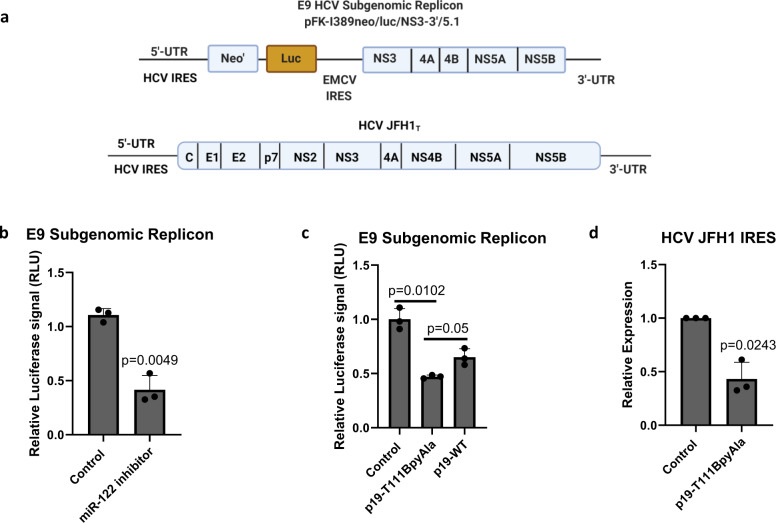
p19-T111BpyAla effect on HCV replication. **a** Schematic representation of the subgenomic HCV replicon (pFK-I389neo/luc/NS3-3′/5.1) used in this study^52^ and HCV JFH1_T_ strain. Created using BioRender.com. **b** Relative luciferase signal upon treatment of E9 cells with either miR-122 antagomir, **c** p19-T111BpyAla or p19-WT. The levels of miR-122 are directly correlated with HCV replication, which is linked to the relative luciferase signal in E9 cells. The effects of both miR-122 antagomir, and p19-T111BpyAla were investigated, with both treatments leading to a similar reduction in HCV subgenomic replication. For **b** and **c**, *n* = 3, and error bars represent mean values ± SD, *p*-values are presented. Unpaired two-tailed t-test was used to evaluate statistical significance. **d** Relative expression of HCV JFH1 IRES in Huh7.5 cells upon treatment with 1 µM p19-T111BpyAla, *n* = 3, error bars represent mean ± SD. *P*-values are presented in the figure. *n* values represent transfections of different cell passages. Control represents no treatment. Unpaired two-tailed t-test was used to evaluate statistical significance.

To investigate the effects of p19-T111BpyAla on the levels of miR-122, we sought to utilize a hepatocellular carcinoma cell line harboring the HCV subgenomic replicon pFK-I389neo/luc/NS3-3′/5.1 (Fig. 6a). This is a replicon system (our version is referred to as the E9 cell line) has been widely used to investigate the replication of HCV in cell culture^52^. Replication of HCV has been shown to be significantly diminished in miR-122 knockout cell line^53^. Thus, we wanted to validate the antiviral role that p19-T111BpyAla can potentially play. We have treated cells with p19-T111BpyAla by directly transfecting the enzyme-Cu^2+^ complex, pre-loaded with copper, into cells or transfecting miR-122 antagomir inhibitor as a control, to validate the effects of the mutant protein on HCV replication. The levels of miR-122 are directly correlated with HCV replication, which is linked to the relative luciferase signal in E9 cells. The effects of both miR-122 antagomir, and p19-T111BpyAla are investigated and correlated to the successful HCV subgenomic replication. Importantly, we were able to show that the designed protein led to almost the same level of HCV inhibition of replication as the levels achieved by the antagomir (Fig. 6b, c). In addition, upon transfecting the enzyme, without the addition of Cu^2+^, we were still able to observe a decrease in luciferase signal, and this can be attributed to the fact that BpyAla can still chelate other divalent metal ions in cells and cellular media used in the assay such as Zn^2+^ (Supplemental Fig. 7)

The basis for the antiviral effects of the antagomir arises from the fact that it binds to the guide strand of miR-122 more tightly than the competing genomic viral RNA. Thus, it prevents binding to and the sequestering of miR-122 by HCV. The p19-T111BpyAla enzyme (loaded with copper) can both sequester miR-122 away from HCV and reduce the overall amount of miRNA through biocatalysis. Thus, with the engineered enzyme, there is the advantage of cleaving the target miRNA reducing its overall levels in the infected cell through catalytic turnover, which was confirmed by quantification of miRNA-122 using Taqman qPCR (Fig. 5c).

In addition to validating the effects of p19-T111BpyAla on HCV subgenomic replication, we wanted to see the effects on Huh7.5 cells infected with JFH1_T_ strain of HCV. Upon HCV infection and then direct protein transfection with 1 µM p19-T111BpyAla-Cu^2+^ (pre-loaded with copper), an inhibition of HCV replication of approximately 50% was achieved (Fig. 6d). Taken together these data support the conclusion that p19-T111BpyAla possesses unique anti-HCV properties by acting as an unnatural endonuclease with substrate selectivity demonstrated towards the proviral miRNA miR-122 (Fig. 6). Taken together, these experiments demonstrated that p19-T111BpyAla has a specific effect on HCV replication in a cellular model system by targeting and cleaving miR-122.

## Discussion

Herein, we show the possibility of engineering endonucleases targeting small RNAs using expanded genetic code techniques to introduce the unnatural amino acid BpyAla site-specifically into p19 protein. We show that upon the addition of copper ions, the engineered p19 VSRS takes on artificial endonuclease activity not found in the wild-type protein or is naturally found in the eukaryotic kingdom^54^. Engineered endonucleases have been previously designed through protein fusions, which in nature can be bulky and could interfere with the functionality and proper folding of the designed enzyme^55^. Thus far, the engineering of RNA endonucleases has been achieved by combining different functional modules of proteins^54^. Similar designs have been applied to DNA binding proteins by introducing a zinc finger DNA binding domain along with an established DNA cleavage domain, with strict selectivity toward a specific DNA sequence^54,55^. However, for the first time, we showed that this approach can be successfully applied specifically to design endonucleases targeting miRNAs, to prevent their function.

An endonuclease based on the viral protein p19 is a good candidate for an RNA endonuclease design that specifically targets miRNAs due to the intrinsic size specificity associated with the protein^26^. We have confirmed that position T111 in the p19 dimer accommodates the introduction of unnatural amino acids as well as copper binding without drastically affecting substrate binding. Position T111 in the p19 dimer has been shown to accommodate different amino acids^31,37^.

As an artificial enzyme, p19-T111BpyAla, should have wide applications in catalytic suppression of the RNA silencing pathway. We demonstrated an application showing that p19-T111BpyAla catalytically degraded human miR-122, which resulted in the downregulation of HCV replication in a cell culture model. RNA therapy and usage of antagomirs, while effective, do have some disadvantages, mainly in costs associated with scale-up and challenges in terms of delivery. In contrast, protein therapeutics have been used and engineered to retain tissue specificity through the employment of protein fusions with tissue-specific peptides, thus holding an advantage over RNA therapeutics^56–58^.

For HCV, it is well established that the virus has evolved a dependence on human miR-122, and those oligonucleotide antagonists (antagomirs) for miR-122 can restrict and even eliminate HCV, even in clinical trials^59,60^. We demonstrated that p19-T111BpyAla was as effective as the corresponding antagomir at reducing miR-122 pools and limiting HCV replication while doing so catalytically and through enzyme-like function. This illustrates the potential for engineered enzymes like p19-T111BpyAla as antiviral strategies in the case where either the virus encodes its viral miRNA or interacts directly with the host’s miRNA as well as in other diseases where miRNAs are disease-causing^10,48,61–63^. Recently, the usage of catalytic enzymes to inhibit viral infections has been investigated, however, it is still a relatively recent concept^27,64,65^.

The p19-T111BpyAla endonuclease shows some selectivity and reasonable potency in different model systems. One challenge is the lack of sequence specificity^17^, which is overcome in the case of HCV replication by the high abundance of the target miRNA. In future iterations, this limitation can be mitigated by utilizing directed evolution techniques to identify other mutants with preferential binding to individual miRNAs of interest, while maintaining the characteristics of p19-T111BpyAla. The current system however can be expressed in specific cells with high expression of a miRNA of interest, in which the engineered endonuclease can preferentially cleave. In this study, miR-122 is the most highly expressed miRNA in liver cells, and thus preferentially targeted.

From the Nanostring profiling experiment, it is evident that other miRNAs exhibit a higher degree of cleavage, which can potentially be attributed to the enhanced binding of p19 mutant to these miRNAs. The differences in affinity could be attributed to the mismatches in the miRNA sequences, which create bulges in the secondary structure of the miRNA, thus affecting the potential binding affinity to p19, depending on the location of the bulge^27,35,46^.

Designing new enzymes based on adding catalytic residues/centers to high-affinity binding motifs has proved challenging mainly due to issues pertaining to product inhibition. Catalytic RNAs, antibodies, and other strategies have a common challenge of lack of substrate turnover due to product inhibition^21–24^. Designing endonucleases based on substrate-binding scaffolds faces the same challenges^25^. However, a key element of our system is the fact that recombinant p19 proteins retain their strong selectivity towards dsRNA of 20–25 nt in length^17^, and high selectivity towards the products of the dicer complex. p19-T111BpyAla at the same time displays little or no affinity towards the ssRNA or 10-11 nt dsRNA products of copper-mediated miRNA cleavage. The low affinity for cleaved products allows for the release of the cleaved product from the enzyme.

In conclusion, we have created the artificial enzyme, p19-T111BpyAla, capable of cleaving and eliminating the products of dicer, siRNAs and miRNAs. This enzyme should have wide applications in catalytic suppression of the RNA silencing pathway. The enzyme also has the potential to be deployed as a therapeutic. It is interesting to think about catalytic systems for targeting human viruses. Our work lays the groundwork for new versions with enhanced targeting.

## Methods

### Expression and purification of p19-WT, p19-K67BpyAla, and p19-T111BpyAla

The coding sequence of p19 retrieved from Carnation Italian Ringspot was cloned in pTriEX-Neo plasmid and subjected to site directed mutagenesis to incorporate amber mutations (TAG) in the positions of interest K67 and T111 using the following primers: forward K67 (5′-AGGAAAGCTGGGTTTCGGGTAGGTTGTCTTTAAGCGCTATC-3′), reverse K67 (5′-GATAGCGCTTAAAGACAACCTACCCGAAACCCAGCTTTCCT-3′), forward T111 (5′-AGCGAGATGCTGCATACTAAACGGAATCGCCGGTC-3′) and reverse T111 (5′-GACCGGCGATTCCGTTTAGTATGCAGCATCTCGCT-3′). Mutagenesis was performed using the Quick-change lighting Site-Directed Mutagenesis kit (Agilent) according to the manufacturer’s protocol. The plasmid was previously constructed in our lab as follows: the CIRV p19 sequence was synthesized by Genescript (Piscataway, NJ) in pUC57, followed by cloning by PCR in pTriEx 4-neo vector (EMD Biosciences, San Diego, CA). this was performed using the following primers: forward (5′-TAAGCCATGGAACGCGCTATCCAAGG-3′) and reverse (5′-CGACTCGAGCTCGCTTTCTTTCTTGAAGG-3′). The PCR product was subsequently digested using NcoI and XhoI (New England Biolabs) and inserted into multiple cloning site of pTriEx 4-neo with a C-terminal 8X histidine tag.

The plasmid encoding BpyAlaRS/tRNA (pEVOL-BpyAla) was a kind gift from Dr. Peter Schultz (The Scripps Research Institute). Transformation of the plasmid encoding BpyAlaRS/tRNA (pEvol-BpyAla) was transformed in OneShot BL21 (DE3) Chemically competent cells (ThermoFisher) and then selected under Chloramphenicol (25 µg/ml). Cells were then re-made competent and transformed with p19 plasmids bearing the appropriate amber mutations, where cells were then plated under the selection of Chloramphenicol and ampicillin (100 µg/ml). Colonies were then selected for protein expression. Cells were then grown in Lysogeny Broth in the presence of the appropriate antibiotics at 37 °C until the optical density of 0.6 was reached. Protein expression was induced using 1 mM IPTG in the presence of 1 mM BpyAla for 4 h at 25 °C. Cells were then harvested and purified using a two-step nickel affinity chromatography and size exclusion chromatography as previously described^66^. Briefly, cells were sonicated and the lysates were loaded onto a HisTrap FF column (GE Healthcare Life Sciences) and eluted using a buffer containing 50 mM Tris pH 8, 300 mM NaCl, 1 mM DTT and 250 mM imidazole. Fractions were further purified using size exclusion chromatography on an S200 column (GE Healthcare Life Sciences) in a buffer containing 20 mM Tris pH 8, 150 mM NaCl, and 1 mM DTT.

### Immunoblotting

Cell pellets were harvested from 1 mL aliquots via centrifugation and lysed in 1x SDS Laemmli sample buffer. Cell lysates were resolved using 12% SDS gel electrophoresis (SDS-PAGE), and then transferred onto a TGX stain-free PVDF membrane (Bio-Rad). Membranes were blocked in 3% BSA in Tris-buffered saline with 0.05% tween (TBS-T). Blot was then incubated with 6xhistag HRP-conjugated antibody (1:5000 dilution; ThermoFisher, MA1-21315-HRP). Membranes were then washed and incubated for 5 min with the ECL Plus Western Blotting System (Bio-Rad). The blot was visualized using ChemiDoc MP imaging system (Bio-Rad). Uncropped blot is presented in Supplemental Fig. 8.

### Substrates

GL2 siRNA cyanine 3 labeled guide strand (5′-Cy3-CGU ACG CGG AAU ACU UCG AUU-3′) and passenger strand (5′-UCG AAG UAU UCC GCG UAC GUU-3′; Sigma-Aldrich). Strands were annealed at 1:1.2 molar ratio in buffer containing 100 mM potassium acetate, 30 mM HEPES pH 7.5, and 2 mM magnesium acetate by heating to 95 °C for 2 min and cooling to 25 °C at a rate of 1 °C/min. The presence of duplexed siRNA was confirmed on a 6% non-denaturing agarose gels (ThermoFisher Scientific).

BHQ-2-GL2-Cy3 substrate: Cyanine 3 labeled guide strand (5′-CGU ACG CGG AAU ACU UCG AUU-Cy3-3′) and passenger strand (5′-UCG AAG UAU UCC GCG UAC GUU-BHQ-2-3′; Dharmacon). Strands were annealed at 1:1.2 molar ratio in buffer containing 100 mM potassium acetate, 30 mM HEPES pH 7.5, and 2 mM magnesium acetate by heating to 95 °C for 2 min and cooling to 25 °C at a rate of 1 °C/min. The presence of duplexed siRNA was confirmed on a 6% non-denaturing agarose gels (ThermoFisher Scientific).

### Electrophoretic mobility shift assay (EMSA)

Varying concentrations of p19 up to 1 µM were incubated for 1 h with 2 nM GL2 siRNA or miR-122 duplexed substrate in 20 mM Tris pH 7, 100 mM NaCl, 1 mM EDTA, 0.02% v/v Tween-20, and 2 mM DTT. Following incubation, samples were resolved on a 6% non-denaturing agarose gels. Visualization of products was performed using a ChemiDoc MP imaging system (Bio-Rad). Data were analyzed using GraphPad Prism v 8.0 and fit to the Eq. (1):

1 $$Y={A}_{\max }\left(\frac{{K}_{{{{{{\rm{D}}}}}}}+{ns}+x}{2{ns}}-\sqrt{{\left(\frac{{K}_{{{{{{\rm{D}}}}}}}+{ns}+x}{2{ns}}\right)}^{2}-\frac{x}{{ns}}}\right)$$

Where *K*_D_ is the dissociation constant, *n* is the number of equivalent sites on the p19 dimer, *s* is the concentration of labeled small RNA, *A*_max_ is the maximal change is fluorescence, and *x* is the concentration of the p19 dimer.

### Denaturing urea-PAGE

For cleavage of the 21 bp GL2 double-stranded siRNA fragment, the reaction was initiated by adding 3-mercaptopropionic acid (final concentration 2.5 mM) to the reaction mixture containing 2 nM Cy3 labeled siRNA (21 bp), 100 nM p19-T111BpyAla, 200 nM CuSO4, in a buffer containing 20 mM HEPES, 100 mM NaCl and 0.02% Tween (pH 7.3) and incubated for 4 h. The reaction mixture RNA was then further purified and concentrated using RNA concentrator kit according to the manufacturer’s protocol (Zymo Research) and the RNA fragments were analyzed by 20% TBE gel containing 8 M urea.

### Fluorescence polarization (FP)

The assay was performed in black 96-well plate to measure FP. Experiments were carried out using excess enzyme concentration [E] = 5000 nM, and Cy3-GL2 siRNA concentration of 200 nM. Reactions were performed in phosphate-buffered saline (PBS) with 10 μM CuSO_4_ and 2.5 mM β-mercaptopropionic acid.

Fluorescence polarization measurements were carried out using SpectraMax i3 (Molecular Devices). Polarization was monitored at 560 nm with excitation at 546 nm. Polarization is expressed as (2) where *I*_V_ and *I*_H_ are the vertically and horizontally polarized emission intensities, respectively.

2 $$P=\frac{{I}_{{{{{{\rm{V}}}}}}}-G{I}_{{{{{{\rm{H}}}}}}}}{{I}_{{{{{{\rm{V}}}}}}}+G{I}_{{{{{{\rm{H}}}}}}}}$$

### Fluorescence turn-on assays

The assay was performed in black 96-well plate to measure fluorescence intensity at excitation of 520 nm, and emission of 570 nm. Experiments measuring multiple turnover reaction rates were carried out using enzyme concentration [E] = 100 nM, and varying excess concentrations BHQ2-GL2-Cy3 siRNA between 150 and 2000 nM. Reactions were performed in phosphate-buffered saline (PBS) with 10 mM DTT, 200 nM CuSO_4_, and 100 µM β-mercaptopropionic acid. V_o_ values are calculated using the initial slopes for the change in product formation over time under different substrate concentrations. Steady-state kinetic parameters were calculated using Graphpad Prism V8.

### Cell culture, RNA Isolation, miRNA profiling, and RT-qPCR

Huh7 cells were seeded in a 6-well plate at 500,000 cells/well. Forty-eight hours post seeding, the cells were lysed using ML lysis buffer. Small RNA fractions were isolated using NucleoSpin miRNA (Macherey-Nagel). 900 ng RNA was then treated with 5 µM protein (p19-WT or T111BpyAla), 10 µM CuSO_4_, 2.5 mM β-mercaptopropionic acid and allowed to incubate for 4 h at 37 °C. 100 attomoles of Cel-miR248 was used as a spike-in oligo control (IDT technologies), serving as an internal monitor to account for any changes caused during RNA purification. RNA was then further purified and concentrated using RNA concentrator kit to remove any protein complexes, according to the manufacturer’s protocol (Zymo Research). Following treatment, relative miRNA levels (miR-122-5p) were quantified using the TaqMan miRNA Assay (Applied Biosystems) Assay ID: 002245, with 10 ng of total RNA used for reverse transcription using the TaqMan MicroRNA Reverse Transcription Kit (Applied Biosystems). For miR-122 quantification, the 2^−ΔΔCt^ method was used to calculate fold changes in expression relative to mock or control treated samples, with RNU6β levels being used for normalization, (Applied Biosystems) Assay ID: 001093.

To analyze miRNA expression on the Nanostring ncounter platform, the RNA treated with either p19-WT or p19-T111BpyAla was prepared as per manufacturer’s protocol for the ncounter human v3 miRNA expression kit. Briefly, 50 ng of RNA was hybridized to the Reporter and Capture probes for 16 h at 65 °C. The prepared samples were loaded onto the nCounter Prep Station to wash the unbound probes and immobilize the samples on the cartridge for analysis and data collection. The target probes were counted using the nCounter Digital Analyzer. The collected data were processed and analyzed using the nSolver analysis software version 3.0. The ratio of expression of miRNAs for p19-T111BpyAla treated samples against p19-WT treated samples were calculated, *n* = 2. Cel-miR248 was used as a spike-in oligo control (IDT technologies), serving as an internal monitor to account for any changes caused during RNA purification. Cel-miR248 levels were used to normalize the quantified RNA levels. Counts below 20 were eliminated from the analysis.

For quantification of *STAT3* mRNA levels upon treatments with p19-T111BpyAla, cells were seeded at 100,000 cells in a 12-well plate. After 24 h, 1 µM p19-T111BpyAla-Cu^2+^ was combined transfection reagent ProteoJuice (Novagen) in opti-MEM media (Gibco) as per manufacturer’s protocol, then supplemented with full media. After another 24 h, cells were lysed for analysis and RNA isolation. RNA isolation from Huh7 cells was done using RNeasy kits (Qiagen) according to manufacturer’s protocol. 250 ng of total RNA was revese-transcribed using superscript II RT kit (life technologies). qPCR was performed using an iCycler (Bio-Rad), using SYBR green SSO Advanced supermix (Bio-Rad) following the manufacturer’s protocol. Primers targeting *STAT3* were used (forward primer 5′-CAGCAGCTTGACACACACGGTA-3′; reverse primer 5′-AAACACCAAAGTGGCATGTGA-3′). Primers targeting the reference gene *RNA18S5* were used (forward primer 5′-GCGATGCGGCGGCGTTATTC-3′; reverse primer 5′-CAATCTGTCAATCCTGTCCGTGTCC-3′).

### E9 subgenomic replicon luciferase assay

1 µM p19-T111BpyAla pre-complexed with CuSO_4_ which the protein was pre-incubated with CuSO_4_ for 30 min on ice, and excess CuSO_4_ was removed by buffer exchange in PBS using 10KDa Amicon Ultra centrifugal filters (Millipore). Samples without CuSO_4_ were also prepared_._ HCV subgenomic replicon pFK-I389neo/luc/NS3-3′/5.1 (E9) cells were seeded at 4 × 10^4^ cells/well in 24-well plates, then either treated with 1 µM p19-T111BpyAla combined with ProteoJuice transfection reagent, or 50 nM hsa-miR-122-5p mirVana miRNA inhibitor (Ambion) transfected using RNAiMax (Life Technologies). Transfections were performed according to manufacturer’s protocol. Cells were lysed after 24 h post transfection in 1× passive lysis buffer (Promega), and the Luciferase assay was carried out using a SpectraMax L luminometer (Molecular Devices).

### JFH-1 infections

Huh7.5 Cells were seeded one day prior to infection in full media (DMEM, 10% FBS, NEAAs) at 20,000 cells/well in 24-well plates. After 24 h, the cells were incubated with the virus at MOI 0.1 in serum-free DMEM for 5 h. 1 µM of p19-T111BpyAla complexed with CuSO_4_ in which the protein was pre-incubated with CuSO_4_ for 30 min on ice, and excess CuSO_4_ was removed by buffer exchange in PBS using 10 kDa Amicon Ultra centrifugal filters (Millipore). p19-T111BpyAla was then combined transfection reagent ProteoJuice (Novagen) in opto-MEM media (Gibco) as per manufacturers protocol, then supplemented with full media and added to the cells. The cells were incubated for 48 h after which they were lysed in RL buffer and RNA was isolated using the Norgen Single Cell RNA purification kit (Norgen Biotek) as per manufacturer’s protocol. Reverse transcription and RT-qPCR were performed with primers targeting the HCV JFH1 IRES (forward primer 5′-GTCTGCGGAACCGGTGAGTA-3′; reverse primer 5′-GCCCAAATGGCCGGGATA-3′). 250 ng of total RNA was revese-transcribed using superscript II RT kit (Life Technologies). qPCR was performed using an iCycler (Bio-Rad), using SYBR green SSO Advanced supermix (Bio-Rad) following the manufacturer’s protocol.

### Inductively coupled plasma mass spectrometry

20 µM p19-WT or p19-T111BpyAla was incubated with 40 µM CuSO_4_ in a total volume of 200 µL 1x phosphate-buffered saline (PBS) buffer and allowed to incubate for 30 min. Excess CuSO_4_ was removed by buffer exchange in PBS using 10 kDa Amicon Ultra centrifugal filters (Millipore). Washes were performed 3 times, to ensure removal of excess copper. All the above solutions were diluted gravimetrically by 10X with 1% HNO_3_, and were measured by Inductively Coupled Plasma Mass Spectrometry using an Agilent 8800QQQ triple quadrupole ICP-MS. Both ^63^Cu and ^65^Cu isotopes were monitored, which showed excellent correlation confirming the absence of any possible polyatomic ion interferences which potentially could have originated from the constituents in the buffer solutions. The measured Cu concentrations in all the measured solutions were well above the limit of detection at 0.09 parts per billion.

### Statistical analysis

Data are presented as the mean of replicates, with error bars representing mean ± standard deviation. Statistical significance was evaluated using two-tailed Student’s t-test and *p* values of less than or equal 0.05 were deemed significant.

### Synthesis of (2-pyridacyl)pyridium Iodide

(2-pyridacyl)pyridium Iodide was synthesized according to previously reported synthetic methods^67^. Iodine (2.0 g, 16 mmol, 0.6 eq.) was dissolved in warm pyridine (7.0 mL, 80 °C) under an argon atmosphere. 2-acetyl pyridine (3.0 mL, 26.7 mmol, 1.0 eq.) was slowly added and the mixture was stirred for 4 h. After cooling, the precipitated solid was collected by filtration and washed with cold pyridine (23 mL). The black solid was then added to boiling ethanol (100 mL) to which 2 spatulas of activated carbon was added. After 1 h, the mixture was filtered via hot filtration. The filtrate was cooled down in an ice bath and the precipitate was collected to yield the desired product as yellow crystals (5.0 g, 15.3 mmol, 57% yield). ^1^H-NMR data matched previously reported data^67^. ^1^H-NMR (300 MHz, DMSO-d_6_) δ, 9.00–9.01 (m, 2H), 8.87–8.89 (m, 1H), 8.70–8.76 (m, 1H), 8.25–8.32 (m, 2H), 8.12–8.17 (m, 1H), 8.06–8.09 (m, 1H), 7.81–7.86 (m, 1H), 6.50 (s, 2H). Raw spectra are presented in the Source data file.

### Synthesis of 5-methyl-2,2′-bipyridine

5-methyl-2,2′-bipyridine was synthesized according to previously reported synthetic methods^67^. In a dry round-bottomed flask, (2-pyridacyl)pyridium iodide (5.0 g, 15.3 mmol, 1.0 eq.) was added with ammonium acetate (2.4 g, 30.6 mmol, 2 eq.). The solid mixture was dissolved in formamide (15 mL) and methacrolein (1.8 mL, 21.4 mmol, 1.4 eq.) was slowly added. The mixture was heated to 75 °C and stirred for 12 h. Once cooled to room temperature, the reaction mixture was poured into saturated NaHCO_3_ and extracted 6 times with 20 mL of EtOAc. The combined organic layers were washed with brine (100 mL), dried with Na_2_SO_4_, and concentrated under reduced pressure. The crude oil was passed through a short silica pad, eluting with 19:1 (DCM:MeOH). The desired product was obtained as a pale yellow oil (1.57 g, 9.2 mmol, 60% yield). ^1^H-NMR data matched previously reported data^67^. ^1^H-NMR (300 MHz, CDCl_3_) δ 8.66 (ddd, *J* = 4.8, 1.8, 0.9 Hz, 1H), 8.50 (dt, *J* = 2.3, 0.8 Hz, 1H), 8.35 (dt, *J* = 8.0, 1.1 Hz, 1H), 8.28 (dd, *J* = 8.1, 0.8 Hz, 1H), 7.79 (td, *J* = 7.8, 1.8 Hz, 1H), 7.67–7.56 (m, 1H), 7.32–7.21 (m, 1H), 2.38 (s, 3H). Raw spectra are presented in the Source data file.

### Synthesis of 5-bromomethyl-2,2′-bipyridine

5-bromomethyl-2,2′-bipyridine was synthesized according to modified previously reported synthetic methods^67^. 5-Methyl-2,2′-bipyridine (2.9 g, 17.3 mmol, 1.0 eq.) was dissolved in CCl_4_ (100 mL) and the solution was degassed via bubbling argon. N-bromosuccinimide (4.0 g, 22.5 mmol, 1.3 eq.) and AIBN (284 mg, 1.73 mmol, 0.1 eq.) were then added to the mixture. A reflux condenser was attached and the mixture was refluxed under inert atmosphere for 3 h. The solvent was then removed under reduced pressure and the crude product was dissolved in 100 mL of CH_2_Cl_2_. 100 mL saturated NaHCO_3_ was poured onto the mixture and it was extracted 3 times with 30 mL of CH_2_Cl_2_. The organic layers were combined, washed with 50 mL of saturated NaHCO_3_, dried with Na_2_SO_4_, and concentrated under reduced pressure. The obtained mixture of mono and bis-brominated products was then dissolved in THF (20 mL) under an argon atmosphere. Diethyl phosphite (0.8 mL, 6.2 mmol, 0.36 eq.) and N,N-diisopropylethylamine (1.1 mL, 6.2 mmol, 0.36 eq.) were added to the mixture and it was stirred for 18 h at room temperature. After full consumption of the dibromide (verified via ^1^H-NMR), the THF was evaporated under reduced pressure and 25 mL of saturated NaHCO_3_ was added to the residue. The mixture was extracted 3 times with 30 mL of EtOAc. The combined organic layers were dried over Na_2_SO_4_ and concentrated under vacuum. The crude product was loaded onto a silica gel column and eluted initially with 200 mL of CH_2_Cl_2_, followed by approx. 500 mL of 200:1 (CH_2_Cl_2_:MeOH). The mono-bromide was obtained as a white solid (2.25 g, 9.0 mmol, 52% yield). ^1^H-NMR data matched previously reported data^67^. ^1^H-NMR (300 MHz, CDCl_3_) δ 8.73–8.63 (m, 2H), 8.40 (dt, *J* = 8.0, 1.1 Hz, 2H), 7.91–7.74 (m, 2H), 7.32 (ddd, *J* = 7.5, 4.8, 1.2 Hz, 1H), 4.54 (s, 2H). Raw spectra are presented in the Source data file.

### Synthesis of 3-(2,2′-bipyridin-5-yl)-L-alanine

Under an inert atmosphere, 5-(bromomethyl)-2,2′-bipyridine (2.3 g, 9.0 mmol, 1.0 eq.), glycine t-butyl ester benzophenone imine (2.80 g, 9.5 mmol, 1.05 eq.) and Maruoka’s catalyst ((11bR)-(–)-4,4-Dibutyl-4,5-dihydro-2,6-bis(3,4,5-trifluorophenyl)-3H-dinaphth[2,1-c:1′,2′-e]azepinium bromide) (20.3 mg, 0.027 mmol, 0.03 eq.) were added to toluene (23 mL) and a 50% aqueous solution of KOH (4.5 mL) was subsequently added. The mixture was stirred for 40 h at −10 °C. The mixture was then diluted with brine (20 mL) and extracted 5 times with diethyl ether (20 mL); the organic layers were combined, dried with Na_2_SO_4_, and concentrated under reduced pressure. The residue was purified by column chromatography on silica gel, eluting with 50:1 (CH_2_Cl_2_:MeOH) to yield the target compound as a yellow oil (3.70 g, 8.0 mmol, 88% yield). ^1^H NMR (300 MHz, CDCl_3_) δ 8.71–8.61 (m, 1H), 8.42 (d, *J* = 2.2 Hz, 1H), 8.36 (d, *J* = 8.0 Hz, 1H), 8.24 (d, *J* = 8.1 Hz, 1H), 7.81 (d, *J* = 1.8 Hz, 1H), 7.65–7.51 (m, 3H), 7.42–7.27 (m, 7H), 6.75 (d, *J* = 7.1 Hz, 2H), 4.17 (d, *J* = 7.4 Hz, 1H), 3.27 (d, *J* = 5.3 Hz, 2H), 1.45 (s, 9H). Raw spectra are presented in the Source data file.

The intermediate product was then dissolved in 6 M HCl (8 mL) and stirred at 60 °C for 12 h. After heating, the solution was cooled down to room temperature and washed 3 times with diethyl ether to remove benzophenone. The aqueous phase was then concentrated and dried under reduced pressure to give the product as a pale white solid (Bpy-Ala•3 HCl) (2.81 g, 8.0 mmol, quant.). Spectral data matched previously reported data^42^. ^1^H NMR (300 MHz, MeOD-*d*_*4*_) δ 8.84–8.80 (m, 2H), 8.67 (dt, *J* = 8.2, 1.1 Hz, 1H), 8.55 (ddd, *J* = 8.2, 7.7, 1.6 Hz, 1H), 8.47 (dd, *J* = 8.3, 0.8 Hz, 1H), 8.21 (dd, *J* = 8.3, 2.2 Hz, 1H), 7.96 (ddd, *J* = 7.6, 5.6, 1.2 Hz, 1H), 4.40 (t, *J* = 6.8 Hz, 1H), 3.41 (qd, *J* = 14.6, 6.8 Hz, 2H). Raw spectra are presented in the Source data file.

### Reporting summary

Further information on research design is available in the Nature Portfolio Reporting Summary linked to this article.

### Supplementary information

Supplementary information

Reporting Summary

### Source data

Source Data

## Data Availability

Data supporting the results and conclusions are available within this paper, the Supplementary Information and Source data files. Source data are provided as Source data file. The Nanostring profiling data generated in this paper have been deposited into NCBI GEO under the accession number: GSE229272. Figures 1b and 3 were made using IRPU 10.2210/pdb6RPU/pdb. Source data are provided with this paper.
