# Author Correction: An unnatural enzyme with endonuclease activity towards small non-coding RNAs

**DOI:** 10.1038/s41467-023-43521-7

**Published:** 2023-11-21

**Authors:** Noreen Ahmed, Nadine Ahmed, Didier A. Bilodeau, John Paul Pezacki

**Affiliations:** Department of Chemistry and Biomolecular Sciences, University of Ottawa, Ottawa, Ontario K1N 6N5 Canada

**Keywords:** Biocatalysis, Synthetic biology, Protein design, Enzymes, RNA

Correction to: *Nature Communications* 10.1038/s41467-023-39105-0, published online 24 June 2023

The original version of this article contained an error in the labels for the gel in Figure 2c. The correct labels are: Lane number 1: WT, Lane number 2: K67, lane number 3: T111, and lane number 4: siRNA and not as previously incorrectly reported Lane number 1: K76, Lane number 2: T111 lane number 3: WT and lane 4: siRNA.

The error has been corrected in the PDF and HTML versions of the Article.

