# Supplementary material for: An unnatural enzyme with endonuclease activity towards small non-coding RNAs
Source: Nat Commun. 2023 Jun 24;14:3777. doi: 10.1038/s41467-023-39105-0 (PMC10290691; doi:10.1038/s41467-023-39105-0)
Supplement: Supplementary file 1 — Supplementary information [file 41467_2023_39105_MOESM1_ESM.docx]

**Supplementary Information**

**An Unnatural Enzyme with Endonuclease Activity Towards Small Non-coding RNAs**

Noreen Ahmed^1^, Nadine Ahmed^1^, Didier A. Bilodeau^1^ and John Paul Pezacki^1,*^

^1^Department of Chemistry and Biomolecular Sciences, University of Ottawa, Ottawa, Ontario, K1N 6N5

^*^Correspondence: [john.pezacki@uottawa.ca](mailto:john.pezacki@uottawa.ca)

Includes:

- Supplementary figure 1-7 and Supplementary table 1


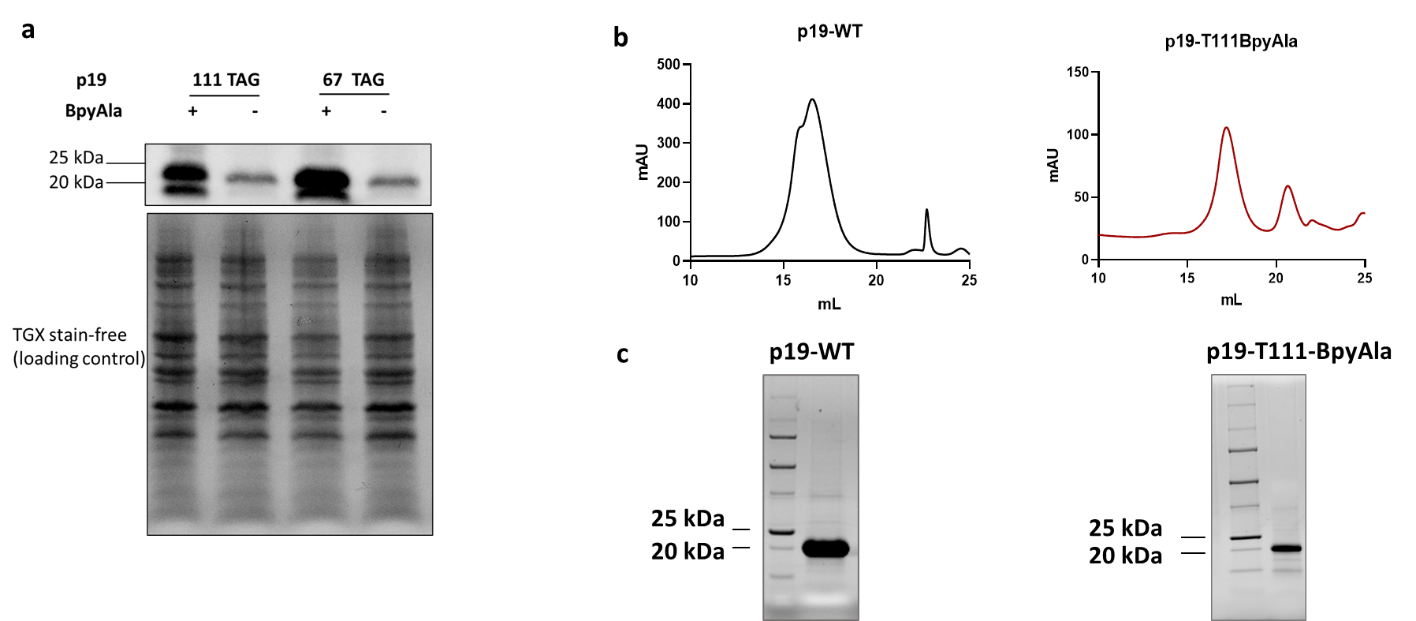


**Supplementary Fig.1. Expression and purification of p19-WT and p19-T111BpyAla purification.** (a) western blot depicting the expression of p19-T111BpyAla and p19-K67BpyAla in *E. coli* cell lysates, where detection was done using an anti-histag antibody. Protein stain represents TGX stain-free loading control. Figure was independently reproduced 3 times (b) size exclusion chromatography (SEC) chromatograms for p19-WT and p19-T111BpyAla on a Superdex 200 column (GE). (c) 5 µL of the concentrated fractions from the SEC were ran on an SDS-PAGE gel to confirm the purification of p19-WT and p19-T111BpyAla. Results were reproduced independently at least 2 times.

**Supplementary Fig. 2. The ICP-MS quantitative analysis of copper concentration bound to p19-WT and mutant p19-T111BpyAla.** Copper concentrations were determined using ICP-MS for samples of p19-WT and p19-T111BpyAla in parts per billion (ppb), for each condition. n=3, n represents three biological replicates, where each replicate represents an independently purified protein batch. Data are represented as mean values ± SD. Unpaired two-tailed t-test was used to evaluate statistical significance.


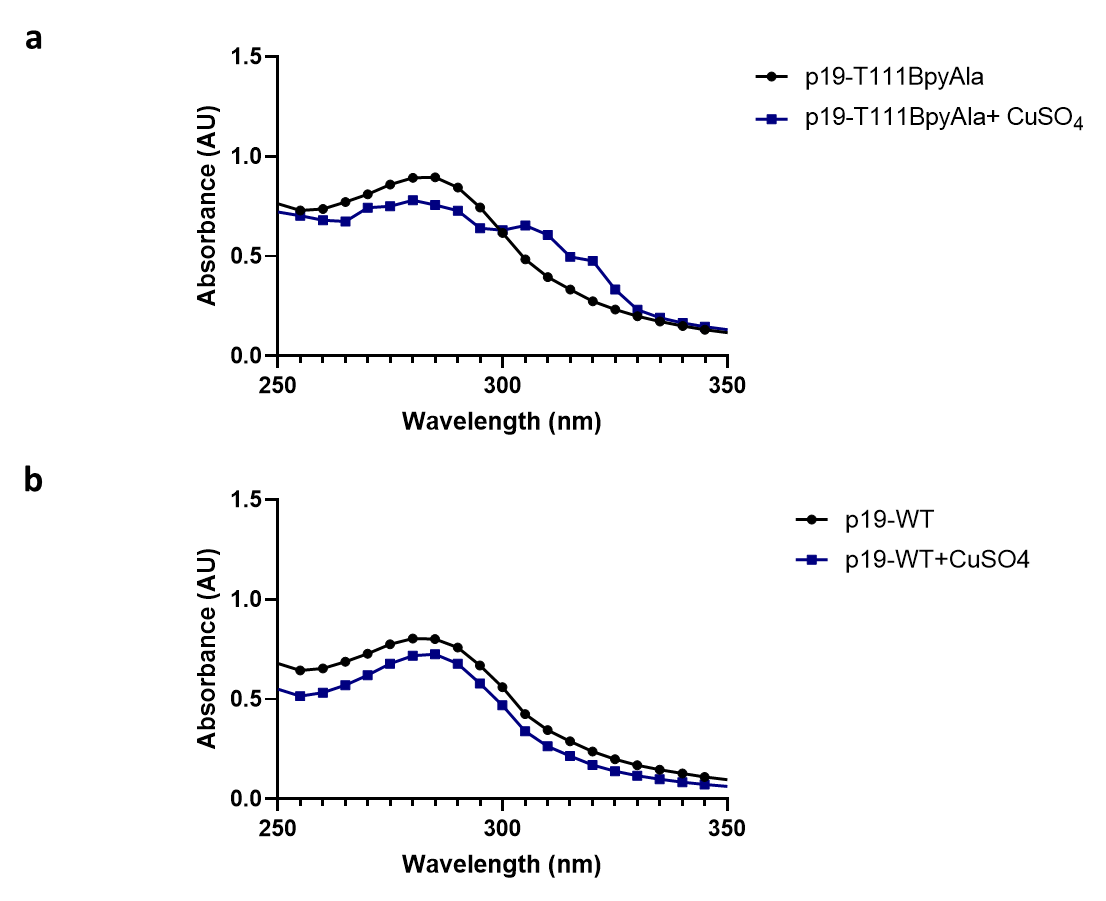


**Supplementary Fig. 3. The UV-Vis absorption spectra of p19 WT and mutant p19-T111BpyAla**. The UV-Vis absorption spectra of 20 μM p19-T111BpyAla mutant upon addition of CuSO4 40μM in 25 mM Tris, 30 mM NaCl (pH = 7.4) showed two new absorption bands at 319 nm and 304 nm, and a decrease in absorption at 283 nm. Spectral changes are in consistent with the red-shift of π – π* transition of the incorporated bipyridyl moiety upon chelation of Cu^2+^ ion.


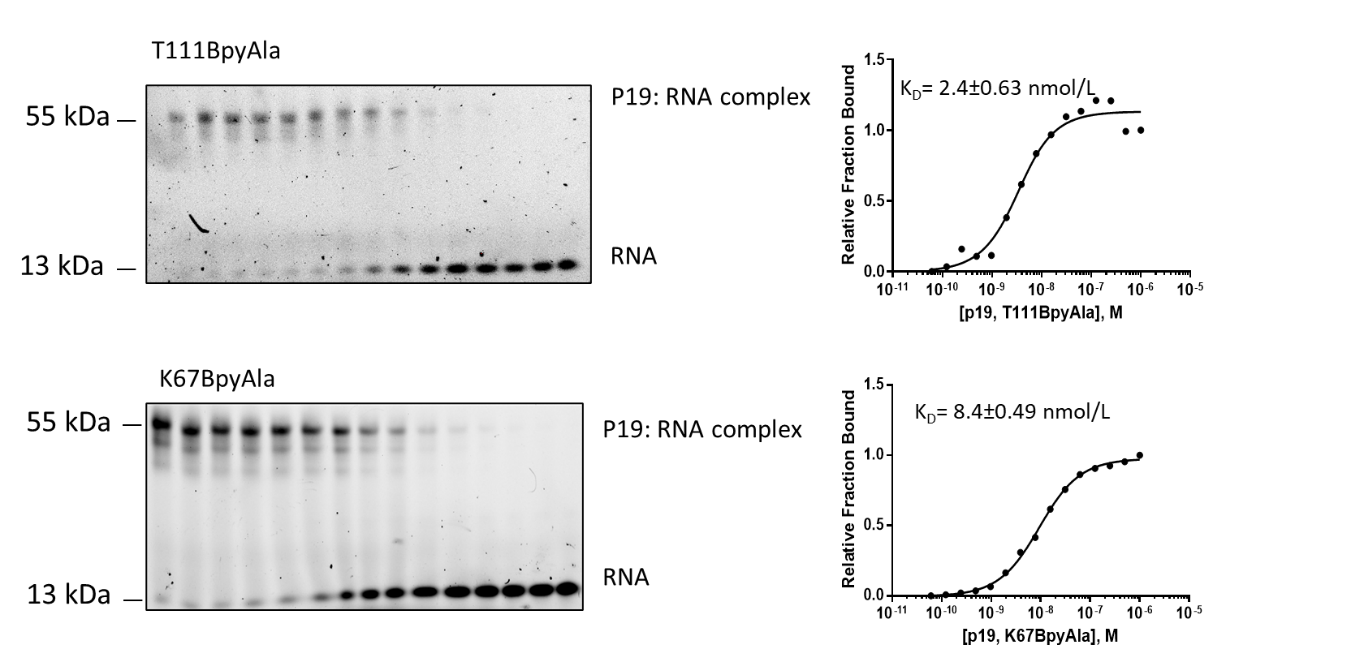


**Supplementary Fig. 4.** **Determination of the Binding affinity of unnatural amino acid p19 mutants.** Binding plot for p19-T111BpyAla and p19-K67BpyAla using electrophoretic mobility shift assay. Gels were reproduced 2 times independently. Binding plots were constructed by varying the concentrations of the protein (0-1 µM), while maintaining the concentration of Cy3-labeled GL2 at 2 nM.


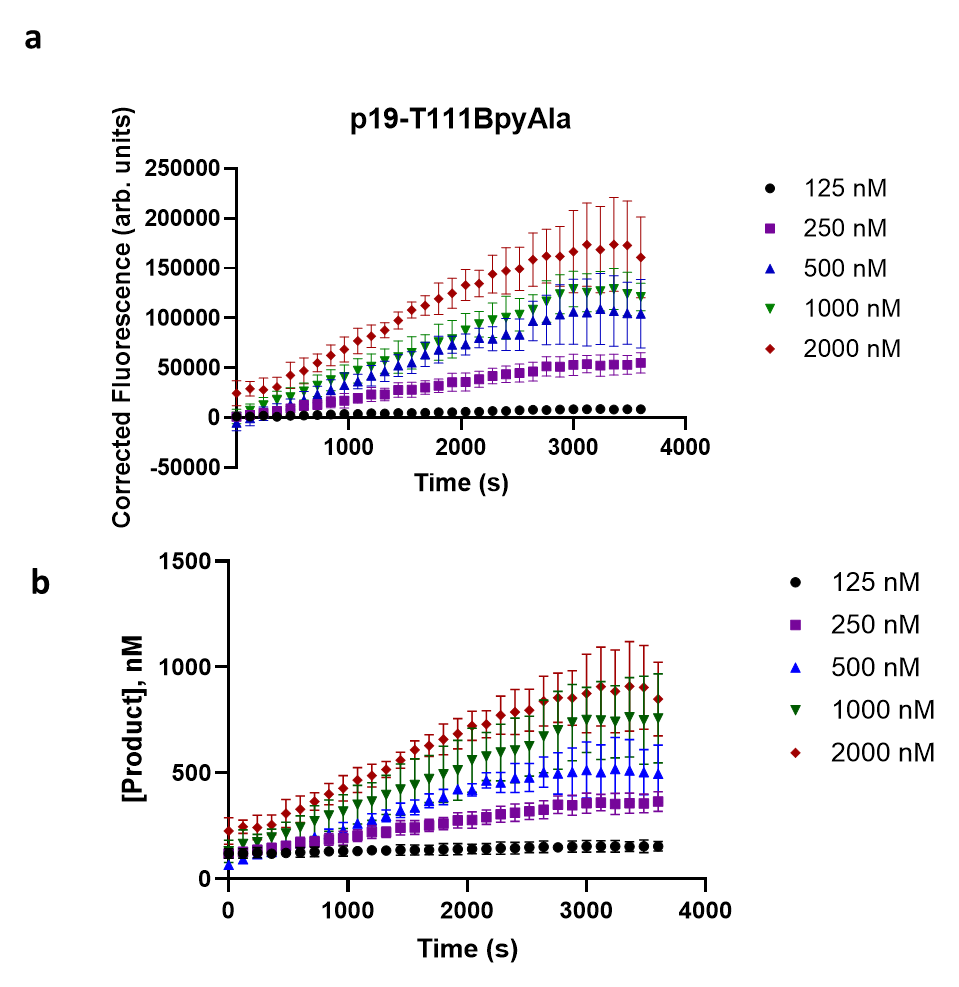


**Supplementary Fig. 5. Time course measurement for varying substrate concentrations.** (a) Fluorescence monitoring over time upon treatment with 100 nM p19-T111BpyAla and varying concentrations of BHQ2-GL2-Cy3 (125-2000 nM) at 37^o^C, excitation: 540 nm and emission; 570 nm. Red represents 2000 nM BHQ2-GL2-Cy3, green 1000 nM, blue 500 nM, purple 250 nM and black is 125 nM. Fluorescence emission represents three independent replicates and error bars represent mean values ±SD. (b) Cleaved product formation over time where increase in fluorescence was converted to product formation in nmol/L. Data represents three independent replicates and error bars represent mean values ±SD. Independent replicates represents proteins from independent purification batches.


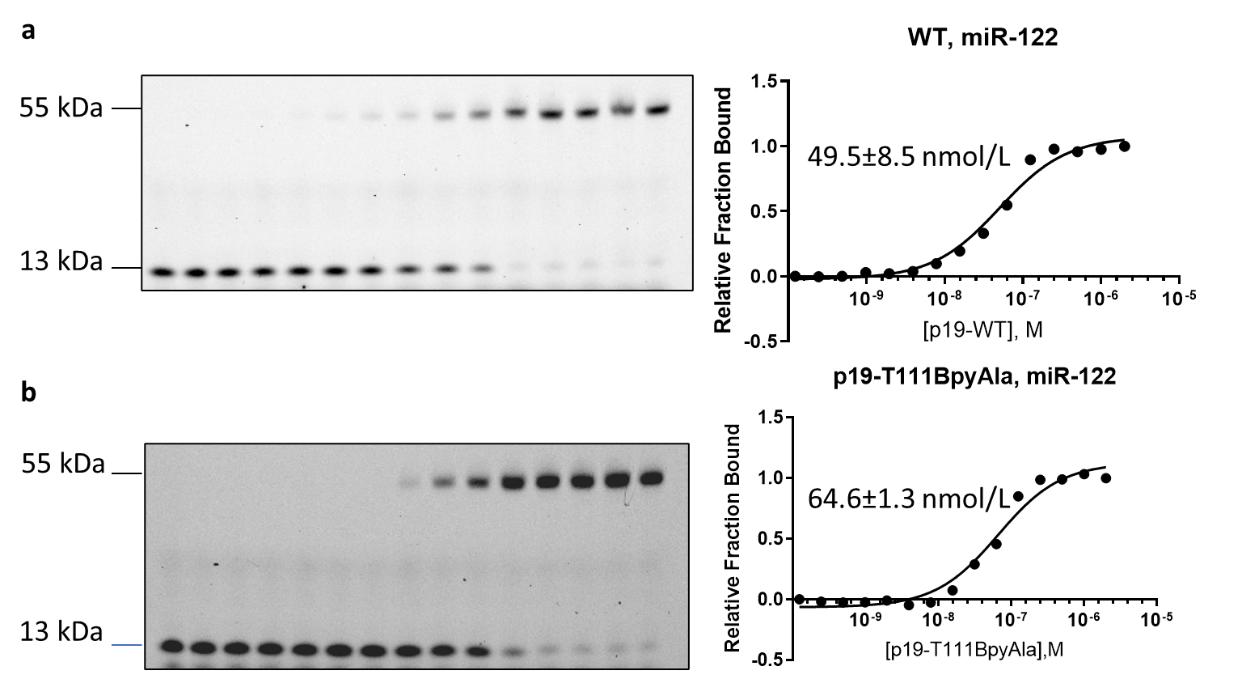


**Supplementary Fig. 6. Determination of the Binding affinity of p19-WT and p19-T111BpyAla towards miR-122.** (a) EMSA gels for p19-WT and p19-T111BpyAla and for p19-K67BpyAla using 2nM Cy3-labeled miR-122. Gels were reproduced 2 times independently (b) Binding plots were constructed by varying the concentrations of the protein (0-2 µM), while maintaining the concentration of Cy3-labeled miR-122 at 2 nM.

**
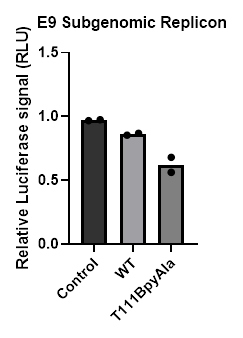
**

**Supplementary Fig. 7. p19-T111BpyAla effect on HCV replication.** Relative luciferase signal upon treatment of E9 subgenomic cells with either p19-T111BpyAla or p19-WT, but no Cu^2+^ was used. The levels of miR-122 are directly correlated with HCV replication, which is linked to the relative luciferase signal in E9 cells n= 2 biological replicates, where protein transfections were performed on two independent cell passages.

**Supplementary Table 1: Summary of miRNAs downregulated upon treatment with p19-T111BpyAla relative to p19-WT.**

Table includes miRNAs which were modulated by at least 1.5-fold during p19-T111BpyAla

treatment of small RNA fractions isolated from Huh7 cell line relative to p19-WT treatments.

| miRNA | Fold Change |
| --- | --- |
| \| hsa-miR-421 \|  \| \| --- \| --- \| \| hsa-miR-10b-5p \|  \| \| hsa-miR-191-5p \|  \| \| hsa-miR-99a-5p \|  \| \| hsa-miR-106a-5p+hsa-miR-17-5p \| \| \| hsa-miR-23a-3p \|  \| \| hsa-miR-122-5p \|  \| \| hsa-miR-93-5p \|  \| \| hsa-miR-194-5p \|  \| \|  \|  \| | \| -1.78 \| \| --- \| \| -1.93 \| \| -1.95 \| \| -1.96 \| \| -1.98 \| \| -1.99 \| \| -2.02 \| \| -2.31 \| \| -2.86 \| \|  \| |

**
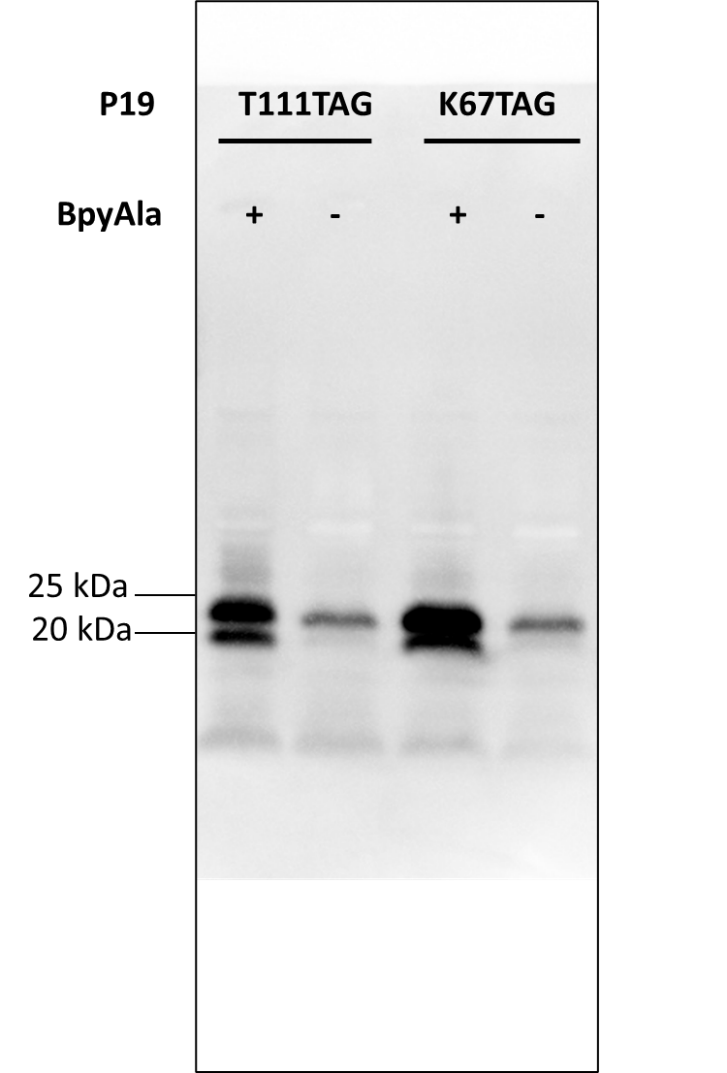
**

**Supplementary Fig. 8. Uncropped BpyAla incorporation blot.** Western blot depicting the expression of p19-T111BpyAla and p19-K67BpyAla in E. coli cell lysates, where detection was done using an anti-histag antibody. Figure was independently reproduced 3 times.
