# Supplementary material for: An unnatural enzyme with endonuclease activity towards small non-coding RNAs
Source: Nat Commun. 2023 Jun 24;14:3777. doi: 10.1038/s41467-023-39105-0 (PMC10290691; doi:10.1038/s41467-023-39105-0)
Supplement: Supplementary file 2 — Reporting Summary [file 41467_2023_39105_MOESM2_ESM.pdf]

nature portfolio

Corresponding author(s): John Paul Pezacki

Last updated by author(s): May 1, 2023

Reporting Summary

Nature Portfolio wishes to improve the reproducibility of the work that we publish. This form provides structure for consistency and transparency in reporting. For further information on Nature Portfolio policies, see our Editorial Policies and the Editorial Policy Checklist.

Statistics

For all statistical analyses, confirm that the following items are present in the figure legend, table legend, main text, or Methods section.

n/a	Confirmed	
☐	☒	The exact sample size (*n*) for each experimental group/condition, given as a discrete number and unit of measurement
☐	☒	A statement on whether measurements were taken from distinct samples or whether the same sample was measured repeatedly
☐	☒	The statistical test(s) used AND whether they are one- or two-sided<br>*Only common tests should be described solely by name; describe more complex techniques in the Methods section.*
☒	☐	A description of all covariates tested
☐	☒	A description of any assumptions or corrections, such as tests of normality and adjustment for multiple comparisons
☐	☒	A full description of the statistical parameters including central tendency (e.g. means) or other basic estimates (e.g. regression coefficient) AND variation (e.g. standard deviation) or associated estimates of uncertainty (e.g. confidence intervals)
☒	☐	For null hypothesis testing, the test statistic (e.g. *F*, *t*, *r*) with confidence intervals, effect sizes, degrees of freedom and *P* value noted<br>*Give P values as exact values whenever suitable.*
☒	☐	For Bayesian analysis, information on the choice of priors and Markov chain Monte Carlo settings
☒	☐	For hierarchical and complex designs, identification of the appropriate level for tests and full reporting of outcomes
☒	☐	Estimates of effect sizes (e.g. Cohen's *d*, Pearson's *r*), indicating how they were calculated

Our web collection on statistics for biologists contains articles on many of the points above.

Software and code

Policy information about availability of computer code

Data collection	AKTA pure Unicorn 6.4.1 was used to collect the FPLC chromatograms. ImageJ was used to analyze the Electrophorectic mobility shift assays.
Data analysis	nSolver analysis software (v 3.0) was used to analyze miRNA expression data according to NanoString-provided guidelines. Graphpad prism v.8 was used to generate plots in the manuscript.

For manuscripts utilizing custom algorithms or software that are central to the research but not yet described in published literature, software must be made available to editors and reviewers. We strongly encourage code deposition in a community repository (e.g. GitHub). See the Nature Portfolio guidelines for submitting code & software for further information.

Data

Policy information about availability of data

All manuscripts must include a data availability statement. This statement should provide the following information, where applicable:

- Accession codes, unique identifiers, or web links for publicly available datasets
- A description of any restrictions on data availability
- For clinical datasets or third party data, please ensure that the statement adheres to our policy

Data supporting the results and conclusions are available within this manuscript the supplementary information.

Field-specific reporting

Please select the one below that is the best fit for your research. If you are not sure, read the appropriate sections before making your selection.

☒ Life sciences ☐ Behavioural & social sciences ☐ Ecological, evolutionary & environmental sciences

For a reference copy of the document with all sections, see nature.com/documents/nr-reporting-summary-flat.pdf

Life sciences study design

All studies must disclose on these points even when the disclosure is negative.

Sample size	Sample sizes were determined on the basis of preliminary experiments and/or experimental designs used in related studies
Data exclusions	no data were excluded
Replication	Fluorescence polarization was replicated 3 times using 3 different protein stocks. kinetic measurements were conducted using 3 different protein stocks and reliably reproduced. Experiments involving protein transfections or treatments were reliably reproduced 3 times using 3 different cell passages.
Randomization	All experiments required mammalian cell transfection or purified protein treatments. Treatments and transfections were tracked with no randomization.
Blinding	The same analysis procedure was maintained regardless of protein treatment or transfection condition. No blinding was used.

Reporting for specific materials, systems and methods

We require information from authors about some types of materials, experimental systems and methods used in many studies. Here, indicate whether each material, system or method listed is relevant to your study. If you are not sure if a list item applies to your research, read the appropriate section before selecting a response.

Materials & experimental systems

n/a	Involved in the study	
☐	☒	Antibodies
☐	☒	Eukaryotic cell lines
☒	☐	Palaeontology and archaeology
☒	☐	Animals and other organisms
☒	☐	Human research participants
☒	☐	Clinical data
☒	☐	Dual use research of concern

Methods

n/a	Involved in the study	
☒	☐	ChIP-seq
☒	☐	Flow cytometry
☒	☐	MRI-based neuroimaging

Antibodies

Antibodies used	6x-His Tag Monoclonal Antibody (HIS.H8) (Invitrogen) MA1-21315-HRP
Validation	This Antibody was verified by the manufacturer. Antibody specificity was demonstrated by the manufacturer through the detection of different targets fused to 6x-His tag in transiently transfected lysates. Relative detection of 6x-His tag was observed across different proteins fused with 6x-His tag. the manufacturer has validated the antibody using the following protocol: Western blot analysis of 6x-His Epitope Tag was performed by loading various amounts of E. coli lysate containing a multi-epitope tagged protein per well onto a 4-20% Tris-HCl polyacrylamide gel. Proteins were transferred to a PVDF membrane and blocked with 5% BSA/TBST for at least 1 hour. The membrane was probed with an HRP-conjugated 6x-His Epitope Tag monoclonal antibody (Product # MA1-21315-HRP) at a dilution of 1:1000 overnight at 4°C on a rocking platform and washed in TBS-0.1% Tween-20. Chemiluminescent detection was performed using SuperSignal West Pico (Product # 34080).

Eukaryotic cell lines

Policy information about cell lines

Cell line source(s)	Huh7, Huh7.5 and Huh7 harboring HCV subgenomic replicon pFK-I389neo/luc/NS3-3′/5.1, E9 cell line are obtained from C.M. Rice (Rockfeller university)
Authentication	The cell lines were not authenticated
Mycoplasma contamination	We have validated that cell lines used in this study are negative for Mycoplasma contamination

Commonly misidentified lines<br>(See ICLAC register)	No commonly misidentified cell lines were used
